# Mechanisms of RNA-induced toxicity in CAG repeat disorders

**DOI:** 10.1038/cddis.2013.276

**Published:** 2013-08-01

**Authors:** R Nalavade, N Griesche, D P Ryan, S Hildebrand, S Krauß

**Affiliations:** 1 German Center for Neurodegenerative Diseases (DZNE), Bonn, Germany; 2 University Bonn, Institute for Pharmacology und Toxicology (AG Prof. Pfeifer), Bonn, Germany

**Keywords:** neurodegeneration, polyglutamine diseases, CAG repeats, RNA–protein interactions, RNA-mediated toxicity

## Abstract

Several inherited neurodegenerative disorders are caused by CAG trinucleotide repeat expansions, which can be located either in the coding region or in the untranslated region (UTR) of the respective genes. Polyglutamine diseases (polyQ diseases) are caused by an expansion of a stretch of CAG repeats within the coding region, translating into a polyQ tract. The polyQ tract expansions result in conformational changes, eventually leading to aggregate formation. It is widely believed that the aggregation of polyQ proteins is linked with disease development. In addition, in the last couple of years, it has been shown that RNA-mediated mechanisms also have a profound role in neurotoxicity in both polyQ diseases and diseases caused by elongated CAG repeat motifs in their UTRs. Here, we review the different molecular mechanisms assigned to mRNAs with expanded CAG repeats. One aspect is the mRNA folding of CAG repeats. Furthermore, pathogenic mechanisms assigned to CAG repeat mRNAs are discussed. First, we discuss mechanisms that involve the sequestration of the diverse proteins to the expanded CAG repeat mRNA molecules. As a result of this, several cellular mechanisms are aberrantly regulated. These include the sequestration of MBNL1, leading to misregulated splicing; sequestration of nucleolin, leading to reduced cellular rRNA; and sequestration of proteins of the siRNA machinery, resulting in the production of short silencing RNAs that affect gene expression. Second, we discuss the effect of expanded CAG repeats on the subcellular localization, transcription and translation of the CAG repeat mRNA itself. Here we focus on the MID1 protein complex that triggers an increased translation of expanded CAG repeat mRNAs and a mechanism called repeat-associated non-ATG translation, which leads to proteins aberrantly translated from CAG repeat mRNAs. In addition, therapeutic approaches for CAG repeat disorders are discussed. Together, all the findings summarized here show that mutant mRNA has a fundamental role in the pathogenesis of CAG repeat diseases.

## Facts

- Several neurodegenerative diseases are caused by the expansion of CAG repeats.
- CAG repeat mRNAs fold into hairpin structures, which increase in size and stability with increasing repeat length.
- CAG repeat mRNAs bind to and sequester diverse proteins.
- RNA-mediated mechanisms have a profound role in neurotoxicity.

## Open Questions

- Are RNA-mediated mechanisms specific to distinct mRNAs or are they common to all CXG repeats?
- Are there additional functions of CAG repeat mRNAs? How do they contribute to neurotoxicity?
- Will strategies to inhibit or neutralize the mutant CAG repeat mRNA species lead to therapeutic approaches for treating CAG repeat disorders?

Short-tandem repeats, such as trinucleotide repeats, represent a substantial portion of the human genome.^1^ One characteristic feature of such repeats is their genetic instability and ability to expand.^2, 3^ Trinucleotide repeat expansions are causative factors for several disorders, including several forms of mental retardation, spinocerebellar ataxias (SCA) and Huntington's disease (HD).^4, 5, 6^ These neurological disorders are caused by the abnormal expansion of CTG, CGG or CAG repetitive elements within the associated genes. CAG repeat diseases are divisible into two groups: the polyglutamine (polyQ) diseases and diseases with causative genes harboring CAG repeats in their untranslated regions (UTRs). PolyQ diseases are characterized by elongated CAG repeats encoding extended polyQ stretches.^4^ These diseases are monogenic and inherited dominantly– and although rare–they constitute the most common form of inherited neurodegenerative disorders. There are currently nine known polyQ diseases (see Table 1). The only similarity between the disease-causing proteins is their extended polyQ tracts. Expansion of the polyQ tract in these proteins results in conformational changes and, eventually, aggregate formation.^4, 7^ The pathogenic role of aggregates has been much debated, with earlier research suggesting that aggregates might contribute to pathogenesis. There have been various proposed mechanisms for polyQ aggregate toxicity. Aggregation may cause a loss of the homeostatic function of the respective proteins as well as the expanded proteins gaining new functions that might be deleterious to the cell. The polyQ-expanded proteins sequester additional proteins (transcription factors, chaperones, and so on) important to the maintenance of cell homeostasis. PolyQ aggregates also negatively influence autophagic mechanisms responsible for the degradation of misfolded proteins. Overall, the importance of aggregates in polyQ toxicity is highlighted by an increase of the protein aggregation propensity and a decrease of the age of disease onset with the CAG repeat number. On the other hand, several recent studies have suggested that the soluble form of the expanded proteins might contribute toward pathogenesis, with the aggregates actually exerting a neuroprotective effect. The role of aggregates and aggregate-prone proteins in pathogenesis has been reviewed extensively elsewhere.^8, 9, 10, 11, 12, 13, 14^ However, the exact molecular and cellular pathways underlying neurodegeneration in polyQ diseases are still largely unknown; further research is needed to clarify the pathogenic roles of aggregates and soluble species. In addition to polyQ protein toxicity, there is increasing evidence that CAG repeat-containing RNA might be directly involved in toxicity. In a *Drosophila* model of SCA3, for example, the interspersal of CAA within the CAG repeat (both encode for Q, but will produce different RNA structures) results in mitigated toxicity, although the protein sequence is unaltered.^15^

The case for direct CAG repeat RNA-mediated toxicity is further bolstered by a second group of CAG repeat diseases, wherein the expanded repeat regions are located in the UTR. In general, the expansion of different nucleotide repeats in the UTR of various genes results in disease development. DM1, for example, is caused by an expanded CTG trinucleotide (OMIM 160 900); SCA8 is caused by an expanded CTG trinucleotide (OMIM 608 768); and FXTAS (fragile X tremor ataxia syndrome) is caused by an expanded CGG trinucleotide (OMIM 300 623). Similarly, the expansion of a CAG repeat in the 5′ UTR is linked to disease development in SCA12 (OMIM 604 326). These observations suggest that toxic mRNA species with expanded CAG repeats contribute significantly to disease development in the absence of polyQ proteins.

In this review, we focus on the various pathogenic modalities of mRNAs with expanded CAG repeats. These include the sequestration of several proteins and transcription factors. In addition, we discuss the effect of expanded CAG repeats on subcellular localization, the transcriptional regulation of CAG mRNA and translation misregulation.

## Three-dimensional Structures of mRNA with CAG Repeat Expansion

To understand the different pathogenic modes of action of mRNAs in CAG repeat diseases, it is important to take a look at the structure of the CAG repeat mRNAs. In this section, we introduce differences in the three-dimensional RNA structure of the mutant *versus* the normal CAG repeat length.

**Figure 1:**
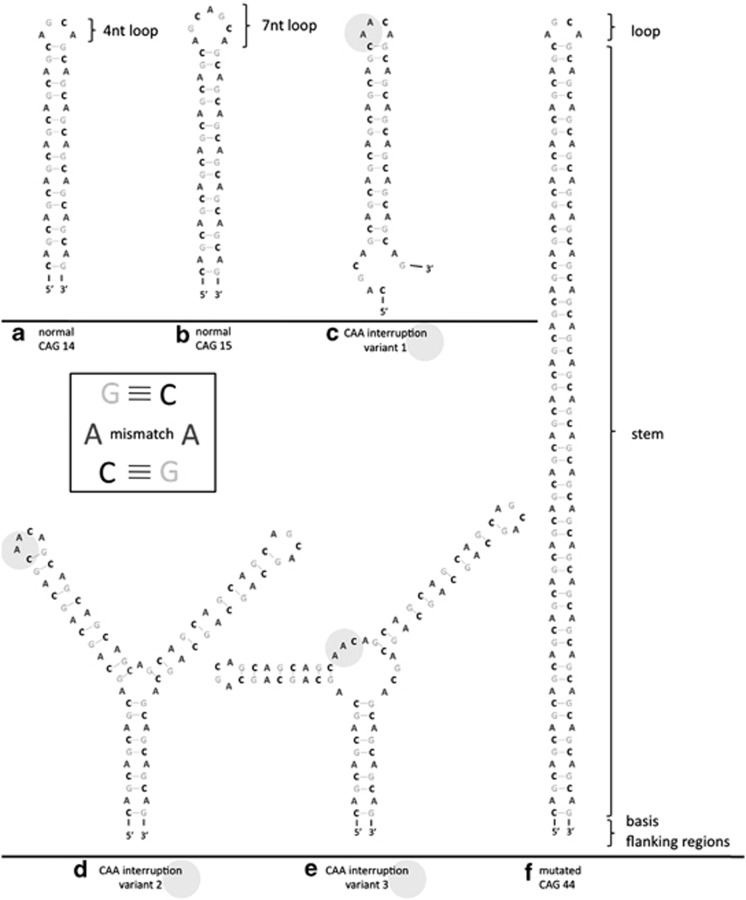
Predicted CAG repeat hairpin structures. Schematic illustration of CAG repeat structures based on *in silico* predictions (using mfold). RNA hairpin formation of CAG repeats: normal length of (**a**) even (CAG14) and (**b**) uneven repeat numbers (CAG15), compared with (**f**) a hairpin formed by pathologically expanded repeat length (CAG44) is shown. In addition, the possible impact of CAA interruptions in the CAG repeat stretch on the hairpin structure is shown in three possible variants (**c**), (**d**) and (**e**)

The secondary structures of CAG and CXG (X is G, A or U) repeat expansions are similar, all having the hairpin formation as a common feature.^16^ Myotonic dystrophy type 1 (DM1) is caused by a CUG expansion and is the best-characterized disease regarding RNA toxicity. As the repeat is in the 3′ UTR of the dystrophia myotonica protein kinase (DMPK) gene, a toxic RNA gain-of-function causes the disease.^17^ This finding has resulted in a plethora of DM1 RNA research. The CUG repeats in the mRNA form hairpins that are stabilized with an increase in the length of the CUG stretch.^18^ Similarly, the *in silico* structural modeling of CAG repeat-containing mRNAs predicts the formation of a hairpin with a stem comprising the CAG repeat region in the Huntingtin (HTT) mRNA.^19^ The CAG repeat region secondary structure consists of a base, a hairpin structure forming the stem and a terminal loop. The stem is formed by repetitive G–C and C–G pairs, followed by an A–A mismatch^16^ (see Figure 1). In a later study, a combination of *in silico* prediction and chemical and enzymatic analyses confirmed the presence of CAG hairpins *in vitro*.^20^ The CAG repeat motif folding is not limited to one structure, but instead varies between several slipped hairpins. Those variants differ in the presence or absence and the length of a single-stranded tail that is composed of the 3′ terminal repeats. Also, the size of the loop can differ between 4 and 7 nucleotides (nt), of which the 4 nt loop is thermodynamically more stable (see Figure 1). The size of this loop depends on the overall repeat number.^16^ Although an even number of CAG repeats mainly results in loops composed of 4 nt, an odd number of CAG repeats leads to the formation of loops with 7 nt. As the hairpin length and stability increase with CAG repeat length, hairpins formed by mutant CAG repeats are more stable than their wild-type counterparts. At the base of the hairpin, specific flanking regions can serve as a natural G–C clamp, stabilizing the hairpin structure.^21^

Silent mutations in CAG repeats can also lead to disease, such as SCA2 (caused by CAG repeats in ataxin 2 (*ATXN2*)), wherein the CAA codons normally interspersed within the CAG repeat are absent in patients, leading to an enhanced uninterrupted CAG repeat. As CAG and CAA both code for Q, there are no resulting amino-acid changes, indicating that mRNA level changes are sufficient for disease development. The structural consequences of CAA interruptions on the hairpin formation are depicted in Figure 1.

Another disease in which codons other than CAG are normally dispersed within the CAG but are absent in patients is SCA1, which is caused by an expanded CAG repeat stretch in the *ATXN1* gene. The *ATXN1* gene in healthy individuals contains a CAG repeat interrupted by CAT triplets (coding for histidine). Loss of these CAT triplets leads to changes in the RNA and the protein level and is associated with disease development. In normal individuals, these interspersed CAT triplets destabilize the hairpin structure, which is then stabilized in patients.^21^

Flanking regions, as well as the hairpin itself, can influence the RNA structure. In an SCA1 transcript model, for example, the flanking regions can form base pairs with each other, leading to a stabilized hairpin.^21^ In contrast, the CAG flanking regions in SCA2 mRNA do not interact with each other. Instead, the 3′ flanking sequence interacts with the 3′ terminal repeats, resulting in several different hairpin structures.^20^

More recently, HTT CAGs have been shown to exhibit the hairpin formation *in vitro*. In addition to the CAG repeat region, the adjacent CCG repeat sequences influence the formation of the CAG hairpin. HTT repeats are structured in a tripartite manner. The base is composed of interacting CAG and CCG repeats, followed by a central motif consisting solely of CAG repeats and a terminal section composed of the fold-back structure from CAG repeats.^22^

High-resolution crystal structures have also proven useful in studying the secondary structure of the CAG repeat region.^23^ Kiliszek *et al.*^23^ used oligonucleotide CAG repeats to investigate the structure using atomic resolution. They discovered that CAG repeats can form three-dimensional α-helical structures, which share some similarities with the three-dimensional structures formed by CUG repeats.

## CAG Repeats in the UTR

Although PolyQ disease-causing genes harbor CAG repeat expansions in their coding regions, leading to expanded Q stretches, similar expansions in UTRs can also result in disease. Two examples with different repeat expansions are SCA8, with a (CTG)_n_ expansion in the *ATXN8OS* gene, and SCA10, containing an (ATTCT)_n_ pentanucleotide repeat expansions in the *ATXN10* gene.^24, 25^ An autosomal dominant disease caused by a CAG expansion in a UTR is SCA12, which is caused by an expansion of a CAG repeat in the 5′ UTR of the *PPP2R2B* gene.^26^ Among other things, the disease is characterized by the action tremor of various body parts and, in later stages, by hyperreflexia, gait ataxia as well as other signs of cerebellar dysfunction and dementia. The disease is rare, with only a few affected people worldwide. Affected individuals seem to have repeats of 51–78 CAGs, where 6–32 are normal.^27^ No polyQ protein translated from the 5′ UTR of the *PPP2R2B* gene has been detected yet,^26^ suggesting that the neuropathology might be directly related to the mRNA. Thus, a toxic gain-of-function of the mutant mRNA because of extended UTR CAG repeats might underlie the observed pathology in this disorder.

Experiments in *C. elegans* further support a direct role of mRNA in the toxicity of disease phenotypes. CAG repeats cloned into the 3′ UTR of a marker protein were toxic in a length-dependent manner in *C. elegans*. Such transgenic nematodes have shortened life spans and reduced motility, with its phenotypic severity increasing concomitantly with the CAG repeat number. The highest repeat numbers were lethal during embryogenesis or at early stages, whereas the shorter CAG repeats did not cause a phenotype.^28^

Although McLeod *et al.*^29^ detected no difference in the external eye appearance of a *Drosophila* model expressing CAG repeats in the 3′ UTR, a subsequent study by Li *et al.*^15^ showed that despite the absence of an eye phenotype, neuronal degeneration was apparent and was caused by RNA toxicity.

Recently, the direct toxicity of mRNA with extended CAG repeats has also been demonstrated in mammals. The expression of RNA containing a long CAG repeat stretch in the 3′ UTR was associated with severe muscle abnormalities. In addition, these mice showed behavioral changes.^30^ It has been suggested that RNA toxicity is tied to the sequestration of proteins by the CAG repeat mRNA, which will be discussed in the next section.

## CAG Repeats Sequester Diverse Proteins

RNA protein complexes are involved in diverse cellular processes, such as transcription, RNA splicing, mRNA transport, translation and mRNA degradation. Aberrant RNA protein complexes may have a crucial role in disease development. In the following section, we will discuss proteins that bind to and can be sequestered by expanded CAG repeat mRNAs and, therefore, might represent pathologic mechanisms in CAG repeat disorders.

### Sequestration of Muscleblind-like 1 (MBNL1) protein induces misregulated alternative splicing

One example of a protein aberrantly binding itself to an mRNA in CAG repeat disorders is the MBNL1 protein with trinucleotide repeat RNA.

The MBNL1 protein is part of the Muscleblind (Mbl) family of proteins, which regulate the alternative splicing of specific target mRNAs, thereby regulating the expression of the specific isoforms of the resulting proteins.^31^ The RNA-binding motif of these proteins is composed of four zinc-finger domains,^32^ with which they bind to their target mRNAs, such as cardiac troponin-T (TNNT2), insulin receptor (IR) pre-mRNA or several others.^33^ Upon binding, MBNL1 can act either as an activator or a repressor of splicing. While inducing IR pre-mRNA exon inclusion, MBNL1 inhibits exon inclusion in the TNNT2 mRNA. MBNL1 binds to the stem-loop structure within the polypyrimidine tract of TNNT2 intron 4 during the spliceosome assembly, where it regulates the exon skipping of exon 5 through competition with the splicing factor U2AF65.^34^ Apart from these specific target mRNAs, Mbl family proteins exhibit a high binding affinity to RNA having trinucleotide repeats beyond a specific length.^32, 35^ This binding ability was elucidated during studies characterizing CUG repeat RNA-binding proteins in the neuromuscular disorder DM1.^36, 37^ DM1 is caused by a CTG trinucleotide expansion in the 3′ UTR of the *DMPK* gene. Studies in DM1 myoblasts and in neurons showed that the expanded CUG repeat mRNAs sequester MBNL1 into nuclear foci.^38, 39^ Aberrant changes in the splicing patterns of several mRNAs have been observed in DM1 and may be due to a loss-of-function mechanism of the MBNL1 protein because of its sequestration into nuclear foci.^40, 41^ This hypothesis has been further backed by the observation that MBNL1 knockout mice exhibit characteristic malformations similar to those of DM1, including the aberrant splicing pattern of several specific RNAs.^42, 43^ Aside from its role in DM1 pathogenesis, the interaction of MBNL1 with CAG repeat RNAs is also of interest. This is unsurprising as the CAG repeat RNAs form a hairpin structure similar to that of CUG repeat RNAs, which is essential for MBNL1 binding. Indeed, it has been observed that expanded CAG repeat mRNAs sequester MBNL1,^22, 35^ leading to the dysregulated alternative splicing of target mRNAs.^44^ Furthermore, the *Drosophila* homolog, Mbl protein, enhances the toxicity of RNA-containing CAG repeat expansions in a *Drosophila* model of SCA3.^15^ Thus, binding and sequestration of MBNL1 represents one mechanism that contributes to RNA toxicity in polyQ expansion disorders.

### Sequestration of nucleolin reduces cellular rRNA

RNAs containing CAG repeats can sequester not only MBNL1, but also other proteins. Tsoi *et al.*^45, 46^ showed that such RNAs are able to bind and sequester the nucleolar protein nucleolin. As nucleolin regulates rRNA transcription, its sequestration reduces rRNA levels, thereby hindering ribosome formation and resulting in the accumulation of unassembled ribosomal proteins. These ribosomal proteins, in turn, bind to and inactivate the p53 inhibitor MDM2 (murine double minute 2), setting off a chain of events ultimately leading to elevated levels of stabilized p53 and apoptosis (this mechanism is termed the Ribosomal Protein-MDM2-p53 pathway).^47^

### Sequestration of transcription factors affect gene transcription

CAG repeat mRNA may also be toxic because of the sequestration of diverse transcription factors. In support of this, untranslated expanded CAG repeat RNAs can alter the transcription of several components of the Akt/Gsk3-β signaling pathway in *Drosophila* via an unknown mechanism.^48^

### Sequestration of DICER leads to the generation of aberrant short silencing RNAs that affect gene expression

The enzyme Dicer is one of the several proteins involved in RNA interference, whereby double-stranded RNA (dsRNA) is processed into short (around 22 nt) RNAs that specifically bind to complementary messenger or viral RNAs, leading to their degradation or to the repression of their translation.^49^ RNA interference is used to silence foreign nucleic acids (e.g., from viruses) and regulate native gene expression (e.g., during development). During this process, dsRNA precursors are processed in the nucleus and exported to the cytoplasm where they bind to Dicer. Dicer belongs to the RNase III family of nucleases and cleaves the long dsRNA into shorter fragments of around 22 nt.^50^ Cleavage products then associate with the nuclease Argonaute–a constituent of the RNA-induced silencing complex–leading to a sequence-specific silencing of the target mRNA expression.^51, 52, 53^

**Figure 2:**
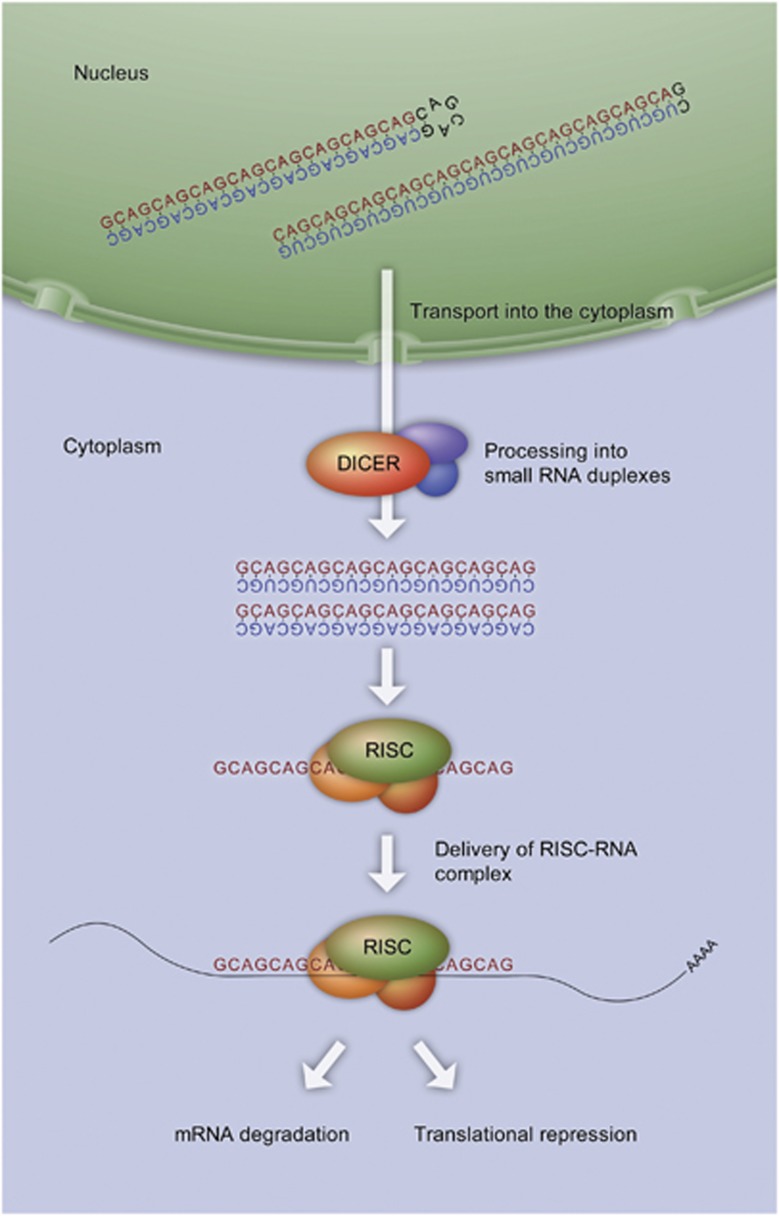
Dicer-dependent production of sCAGs. The enzyme Dicer cleaves long double-stranded CAG repeat RNA into shorter fragments of 22 nt, termed sCAGs. These cleavage products associate with the RNA-induced silencing complex (RISC), which separates the strands. RISC loaded with single-stranded RNA translocates to target mRNAs having complementary sequences. Binding of the loaded RISC to the target mRNA results in the translational inhibition of the target mRNA

Hairpins formed by the triplet repeat expanded RNAs can resemble structures formed by dsRNA, thereby acting as substrates for Dicer. This phenomenon was reported for CGG repeat RNA in fragile X syndrome.^54^ Above a certain length, all CNG repeats are cleaved by Dicer, leading to the formation of 21 nt repeat-containing fragments.^55^ These fragments can bind to complementary transcripts and downregulate their expression via an RNA interference-like mechanism, as explained above. For example, the generation of 21 nt long CAG repeat fragments (or sCAGs) from expanded CAG repeat RNA is observed in HD cell models. The sCAG generation correlates with the CAG repeat number. sCAGs are able to bind to CTG repeat-containing genes through the RNA interference machinery,^56^ which in turn leads to a misregulated expression of CTG repeat-containing genes (Figure 2).

### Antisense transcription generates aberrant short silencing RNAs that affect gene expression

Antisense transcription is the generation of transcripts from the strand opposite to that acting as a template for the generation of protein coding mRNAs. Antisense transcription occurs in the mammalian transcriptome^57^ leading to the generation of antisense (often non-coding) RNAs that have several roles (reviewed in Faghihi and Wahlestedt^58^), including the regulation of sense mRNA transcription and stability.^59^

Antisense transcription is known to occur in SCA8 and HD-like 2 (HDL2), which are characterized by CTG/CAG repeats. In SCA8, two genes encompassing the repeats are expressed: *ATXN8*, on the sense strand, codes for an expanded polyQ protein, whereas *ATXN8OS*, on the antisense strand, expresses a CUG repeat RNA that is non-coding.^60^ In HDL2, the repeat expansion occurs at the locus that includes the gene Junctophilin-3 (*JPH3*), where a CTG repeat is expanded on the sense strand. The expression of a CAG repeat transcript from the antisense strand produces a toxic polyQ protein in a mouse model,^61^ whereas in HDL2 patients, polyQ proteins could not be detected despite the expression of CAG repeat RNA.^62^ Apart from direct polyQ protein toxicity, antisense CAG repeat RNA might itself directly contribute to the disease. This contribution might be imparted through the RNA interference machinery. For example, co-expression of expanded CAG transcripts, together with expanded CTG repeat transcripts in a *Drosophila* model of triplet repeat diseases, enhances toxicity primarily through the generation of sCAGs that misregulate gene expression via an RNA interference-like mechanism, as explained above.^63, 64^

### PKR (dsRNA-dependent protein kinase)

PKR is a ubiquitously expressed serine/threonine protein kinase activated by different mechanisms, including interferon, dsRNA, cytokines, growth factors and stress.^65^ Initially, it was described in viral response, where the viral dsRNA triggers the activation of PKR via autophosphorylation. This leads to an inhibition of protein synthesis and transcription of inflammatory genes. Direct interaction via phosphorylation has only been reported for eIF-2α, although other pathways seem to be indirectly regulated by PKR.^65^ Tian *et al.*^66^ demonstrated that PKR binds to CUG repeats, with the binding affinity increasing with the repeat length. They concluded that either sequestration or activation of PKR might have a role in toxicity. Peel *et al.*^67^ showed that PKR binds to mutant HTT RNA transcripts as well. Performing pull-down assays with human brain extracts, they showed that the phosphorylated form of PKR is bound to mutant HTT mRNA. In addition, they could detect increased phospho-PKR immunostaining in areas associated with HD in post-autopsy human HD samples and brain tissue from HD mouse models. In brain tissue from HD patients, an increase of phosphorylated PKR could also be shown in hippocampal neurons. Furthermore, a nuclear localization as well as aggregation of pPKR is observed.^68^

## Effect of Expanded CAG Repeats on Subcellular Localization, Transcription and Translation of CAG Repeat mRNA

While the previous section focused on a variety of proteins sequestered by expanded CAG repeat mRNAs and the consequences of this, in the next section, we will focus on how the CAG repeat mRNA itself is differentially regulated depending on the repeat lengths.

### Transcriptional regulation of CAG mRNA

Recently, cellular factors necessary for the efficient transcription of genes with CAG repeats have been identified. Liu *et al.*^69^ discovered the transcription factor Spt4/Supt4h when screening for modifiers of the toxicity of proteins carrying expanded polyQ stretches. Spt4/Supt4h is required only for the transcription of long CAG repeat stretches. The siRNA-mediated knockdown of Supt4h significantly reduced the levels of HTT carrying expanded CAG repeats, leaving the short CAG repeat-containing HTT levels unchanged. Strikingly, this study suggests that the pharmacological inhibition of transcription factors could be a promising strategy to specifically reduce the expression of mutant polyQ proteins, and at the same time leave the expression of the functional wild-type HTT unaffected.

### Increased translation of CAG repeat mRNAs

**Figure 3:**
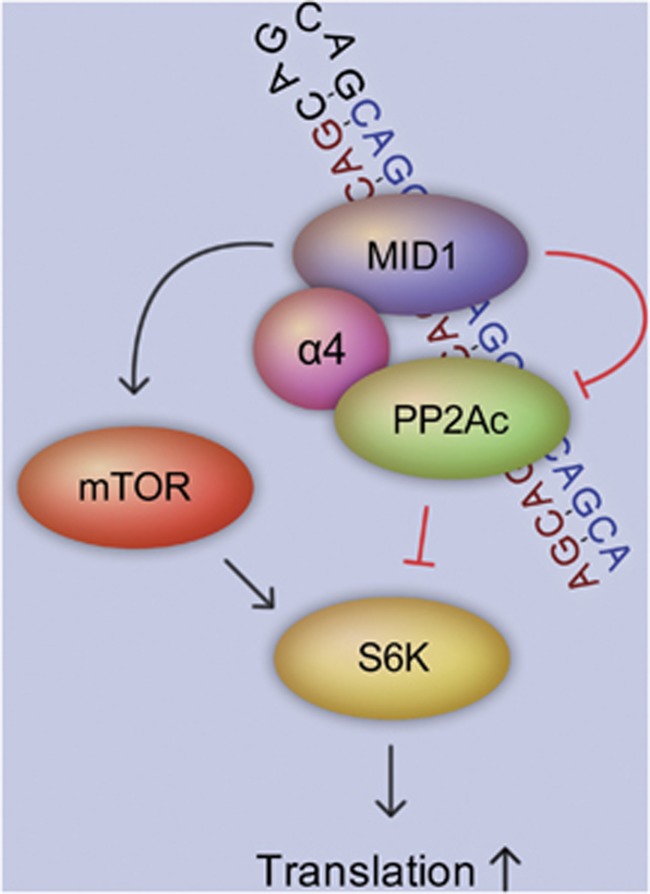
Translation of expanded CAG repeat mRNAs is regulated via the MID1 protein complex. The MID1 protein mediates the binding of the translational regulator S6K to expanded CAG repeats. PP2A and mTOR control the phospho-dependent activity of S6K. MID1 is an inducer of mTOR and an inhibitor of PP2A and, therefore, indirectly stimulates translation via S6K

Another way that expanded CAG repeats in the mRNA induce an abnormal regulation is via targeting proteins for protein synthesis. We have shown recently that HTT mRNAs carrying expanded CAG repeats bind to a protein complex containing the MID1 protein, the catalytic subunit of protein phosphatase 2A (PP2Ac) and 40S ribosomal S6 kinase (S6K), a target of mTOR kinase and PP2A.^70^ MID1 is an ubiquitin ligase that ubiquitinates PP2Ac and thereby targets it for degradation via the proteasome. Therefore, MID1 is a negative regulator of PP2A.^71^ Through this negative regulatory influence on PP2A activity, MID1 also controls the activity of the mTOR kinase.^72^ Both enzymes, mTOR and PP2A, have important roles in the regulation of protein translation by controlling the phosphorylation and activity of several translation regulators, such as S6K. Phospho-activated S6K phosphorylates and enhances the activity of its targets, elF4B (eukaryotic translation initiation factor 4B) and ribosomal protein S6, which in turn unwind and linearize the 5′ UTR of their target mRNAs and promote ribosome binding and translation initiation. Apart from this, the MID1 complex contains polyribosomes and several translation factors and is able to bind to and induce the translation of mRNA.^73, 74^ HTT mRNA binds to the MID1 protein complex via its CAG repeat in a length-dependent manner, with expanded–as opposed to nonpathalogical–CAG repeats binding essentially more protein. This sequestration of the MID1 complex results in an enhanced translation of mutant HTT mRNA, which in turn leads to the accumulation of mutant HTT protein (Figure 3).^70^ Therefore, the inhibition of the MID1 complex could be a successful strategy to suppress the translation of toxic polyQ protein in diseases like HD.

### RAN translation might contribute to the pathology of polyQ diseases

Repeat-associated non-ATG translation (RAN translation) is another translation-level pathogenic method of CAG repeat-containing mRNAs. In experiments using ATXN8 constructs containing expanded CAG repeats lacking the start codon, Zu *et al.*^75^ discovered that RAN translation is initiated in long hairpin structures formed by CAG repeats. RAN translation results in the expression of additional proteins harboring expanded polyQ, polySerine (S) or polyAlanine (A) tracts. Therefore, mRNAs with expanded CAG repeats could induce toxicity on an additional level: by the production of polyQ, polyS or polyA proteins, all of which might be linked to disease pathology.^75, 76^

### Subcellular localization and nuclear export of CAG repeat mRNAs

Normally, mRNA transcripts deliver the message from the DNA sequence to the cytoplasm to induce protein translation. In neurological diseases with CUG expansions, for example, in DM1, the mutant mRNA is retained in the nucleus. Here, it binds in excess to proteins like MBNL1. The robust sequestration of proteins to these nuclear RNA foci has been proposed to be a toxic mode of action of mRNA molecules (see above).^77^

Similarly, nuclear aggregates containing mRNAs with expanded CAG repeats, for example, HTT or ATXN3 mutant mRNAs, have been observed.^22, 78^ Although an abundance of these foci per nucleus increases with repeat length,^30^ no difference in the percentage of foci-positive nuclei has been observed.^77^ A potential mechanism for the nuclear retention of CAG mRNAs has been recently described for ATXN3. In a *Drosophila* model expressing an ATXN3 cDNA with 78 CAG repeats, U2AF50 (or its human ortholog U2AF65), a protein involved in nuclear export, binds to mRNAs with expanded CAG repeats. Of note, in immunoprecipitation experiments, the authors detected no binding of U2AF65 to shorter (27 CAG) repeats. Furthermore, in symptomatic mouse models of polyCAG repeat disorders, the U2AF65 level declines. This leads to a decrease of the nuclear export and to the nuclear accumulation of mRNAs with expanded CAG repeats.^79^

## Therapeutic Approaches

**Figure 4:**
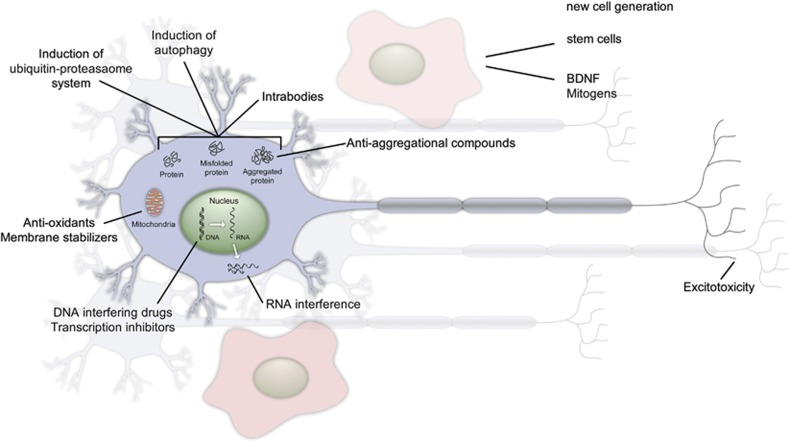
Therapeutic approaches for the treatment of HD and other CAG repeat disorders. Treatments of CAG repeat diseases range from the reduction of aggregate-prone proteins over mitochondrial manipulation to targeting DNA and using RNA interference in the cell. Several of these methods are already in clinical trials, including, for example, anti-oxidants. The generation of new cells using stem cell therapy, also analyzed in clinical trials, and the induction of differentiation through BDNF is another approach for therapy

Traditional treatments for HD and other CAG repeat-containing neurodegenerative diseases are only palliative in nature. The therapy for HD consists of a combination of antidepressants, antipsychotic drugs for movement disorders as well as for psychiatric symptoms and/or mood stabilizers in addition to speech and physical therapy. Recent research has led to a wide variety of new therapeutic approaches that are in different test phases (see Figure 4). Much of the current research is focused on the most common polyQ disease, HD.

One possible approach to the treatment is the reduction of mutant protein. To lower the mHTT level, several different studies focus on targeting the misfolded protein,either by using anti-aggregation compounds,^80, 81^ intracellular antibodies,^82, 83, 84, 85^ inducing autophagy,^14, 86, 87, 88, 89, 90^ or by increasing the ubiquitin–proteasome system-mediated clearance.^87, 91, 92, 93^

Other approaches aim to replace lost neurons. Here, therapeutic approaches using fetal striatal cells or medium spiny neurons derived from embryonic stem cells have been used in animal models and HD patients.^94, 95, 96^ Furthermore, treatments that themselves trigger adult neurogenesis are being tested.^80, 95, 97, 98, 99, 100^

Mitochondrial dysfunction and oxidative stress contribute to neurodegeneration, and treatments involving their modulation are currently under investigation.^101, 102, 103^

Yet another approach focuses on balancing transcriptional dysregulation in HD.^80, 104, 105^

Most of these approaches solely target either the mutant protein or mechanisms that take place after the polyCAG repeat mRNA has been transcribed, therefore not affecting RNA toxicity. Instead, direct targeting of mutant HTT RNA would have the advantage of interfering at the beginning of the pathogenesis. This might be accomplished with RNA interference, which allows the specific targeting of the mutant HTT transcript.^80, 106, 107, 108, 109, 110^

## Concluding Remarks and Future Directions

There are multiple components underlying CAG expansion mediate disorders, which involve aggregate-prone polyQ proteins as well as untranslated CAG repeat expansions at the mRNA level.

**Figure 5:**
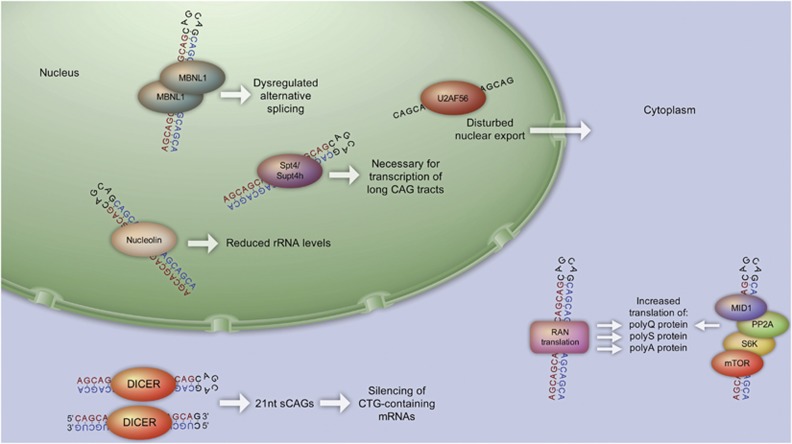
Neurotoxic mechanisms of CAG repeat mRNA. These include nuclear events like sequestration of the MBNL1 protein, which result in misregulated alternative splicing or the sequestration of other proteins like nucleolin, resulting in reduced rRNA levels. With increasing lengths of the CAG repeat, nuclear export becomes inhibited. In the cytosol, there is a Dicer-dependent production of sCAGs from either CAG repeat hairpins or from dimers of the CAG sense–CUG antisense transcripts. These sCAGs have the potential to silence CTG repeat-containing mRNAs. In addition, translational misregulation of polyCAG mRNAs occurs, including RAN translation of polyQ, polyA and polyS proteins from the CAG repeat mRNA molecule, as well as the increased translation of expanded CAG repeat mRNAs via the MID1 protein complex

The studies highlighted above demonstrate that CAG repeat expansions in mRNAs can contribute to neurotoxicity in CAG repeat expansion disorders at several levels (Figure 5). A number of different RNA-binding proteins are recruited to the expanded CAG repeat motifs, leading to a disruption of several pathways involving alternative splicing, reduction of cellular rRNA, misregulated gene transcription and production of aberrant short silencing RNAs. Future studies are needed to explore additional roles of CAG repeat mRNAs and to further investigate their contribution to neurotoxicity.

Furthermore, we have summarized studies demonstrating the differential subcellular localization, transcriptional and translational regulation of the expanded mutant CAG repeat mRNAs, compared with the wild-type repeats. Strategies to inhibit or neutralize the mutant mRNA species could be a promising therapeutic approach for treating CAG repeat disorders. From the studies highlighted above, two putative therapeutic approaches become apparent: the inhibition of Spt4/Supt4h to repress the transcription of expanded CAG repeat mRNAs and the inhibition of the MID1 complex to repress the translation of expanded CAG repeat mRNAs.

In this review article, we have summarized different pathomechanisms involving mRNAs with expanded CAG repeats. These studies rewrite the older textbook supposition that mRNAs act only as messengers, shuttling genetic information to the protein level, where the eventual units of biological activity and disease causation reside. Molecular insight into the pathology of CAG repeat expansion disorders shows that the mutant mRNA has a fundamental role in the pathogenesis of these diseases. This conclusion is an important realization that in the future it will have to influence both diagnostic as well as therapeutic avenues.

## Figures and Tables

**Table 1:** PolyQ diseases

**Disease**	**Gene**	**Symptoms**	**Normal repeat number**	**Mutant repeat number**	**OMIM**	**Estimated prevalences (orphanet; of note there are significant geographical and ethnic variations)**
HD	HTT	Chorea, dystonia, incoordination, cognitive decline and behavioral difficulties	9–36	<37	143 100	Approximately 1:10 000
SCA1	ATXN1	Cerebellar ataxia, supranuclear ophthalmoplegia, pyramidal or extrapyramidal signs, mild dementia, and peripheral neuropathy	8–44	39–83	164 400	Approximately 1–2:100 000
SCA2	ATXN2	Cerebellar ataxia, supranuclear ophthalmoplegia, pyramidal or extrapyramidal signs, mild dementia, and peripheral neuropathy	13–31	32–79	183 090	Approximately 1–2:100 000
SCA3/MJD	ATXN3	Cerebellar ataxia, spasticity, ocular movement abnormalities	>44	52–86	109 150	Approximately 1–2:100 000
SCA6	CACNA1A	Cerebellar ataxia, dysarthria, visual disturbances, dysphagia	4–18	19–33	183 086	Approximately 0.6–3:1 000 000
SCA7	ATXN7	Cerebellar ataxia with pigmentary macular degeneration, ophthalmoplegia, pyramidal or extrapyramidal signs, deep sensory loss, or dementia	4–35	37–306	164 500	Approximately 0.6 : 1 000 000
SCA17	TBP (TATA box-binding protein)	Ataxia, pyramidal and extrapyramidal signs, cognitive impairments, psychosis, and seizures	25–44	47–63	607 136	Approximately 0.47–1.6 : 1 000 000
DRPLA	ATN1 (atrophin-1)	Myoclonic epilepsy, dementia, ataxia, and choreoathetosis	29–42	47–55	125 370	Approximately 1 : 208 000
SBMA	AR (androgen receptor)	Spinal and bulbar muscular atrophy	10–36	38–62	313 200	Approximately 1 : 30 000 male births

The nine polyQ diseases, with the respective disease-causing gene, the number of normal or expanded CAG repeats as well as phenotypic characteristics of the patients are listed

## Abbreviations

SCA: spinocerebellar ataxias
Q: glutamine
polyQ: polyglutamine
HTT: Huntingtin
DM1: myotonic dystrophy type 1
ATXN: ataxin
HD: Huntington's disease
nt: nucleotides
PP2A: protein phosphatase 2A
S6K: 40S ribosomal S6 kinase
